# Altered Brain Functional Connectivity in Betel Quid-Dependent Chewers

**DOI:** 10.3389/fpsyt.2017.00239

**Published:** 2017-11-20

**Authors:** Xiaojun Huang, Weidan Pu, Haihong Liu, Xinmin Li, Andrew J. Greenshaw, Serdar M. Dursun, Zhimin Xue, Zhening Liu

**Affiliations:** ^1^Mental Health Institute of the Second Xiangya Hospital, Central South University, Changsha, China; ^2^The China National Clinical Research Center for Mental Health Disorders, National Technology Institute of Psychiatry, Key Laboratory of Psychiatry and Mental Health of Hunan Province, Changsha, China; ^3^Medical Psychological Institute, Second Xiangya Hospital, Central South University, Changsha, China; ^4^Mental Health Center of Xiangya Hospital, Central South University, Changsha, China; ^5^Department of Psychiatry, University of Alberta, Edmonton, AB, Canada

**Keywords:** betel quid, resting-state, functional connectivity, independent component analysis, network

## Abstract

**Background:**

Betel quid (BQ) is a common psychoactive substance worldwide with particularly high usage in many Asian countries. This study aimed to explore the effect of BQ use on functional connectivity by comparing global functional brain networks and their subset between BQ chewers and healthy controls (HCs).

**Methods:**

Resting-state functional magnetic resonance imaging (fMRI) was obtained from 24 betel quid-dependent (BQD) male chewers and 27 healthy male individuals on a 3.0T scanner. We used independent component analysis (ICA) to determine components that represent the brain’s functional networks and their spatial aspects of functional connectivity. Two sample *t*-tests were used to identify the functional connectivity differences in each network between these two groups.

**Results:**

Seventeen networks were identified by ICA. Nine of them showed connectivity differences between BQD and HCs (two sample *t*-tests, *p* < 0.001 uncorrected). We found increased functional connectivity in the orbitofrontal, bilateral frontoparietal, frontotemporal, occipital/parietal, frontotemporal/cerebellum, and temporal/limbic networks, and decreased connectivity in the parietal and medial frontal/anterior cingulate networks in the BQD compared to the HCs. The betel quid dependence scale scores were positively related to the increased functional connectivity in the orbitofrontal (*r* = 0.39, *p* = 0.03) while negatively related to the decreased functional connectivity in medial frontal/anterior cingulate networks (*r* = −0.35, *p* = 0.02).

**Discussion:**

Our findings provide further evidence that BQ chewing may lead to brain functional connectivity changes, which may play a key role in the psychological and physiological effects of BQ.

## Introduction

**Figure 1:**
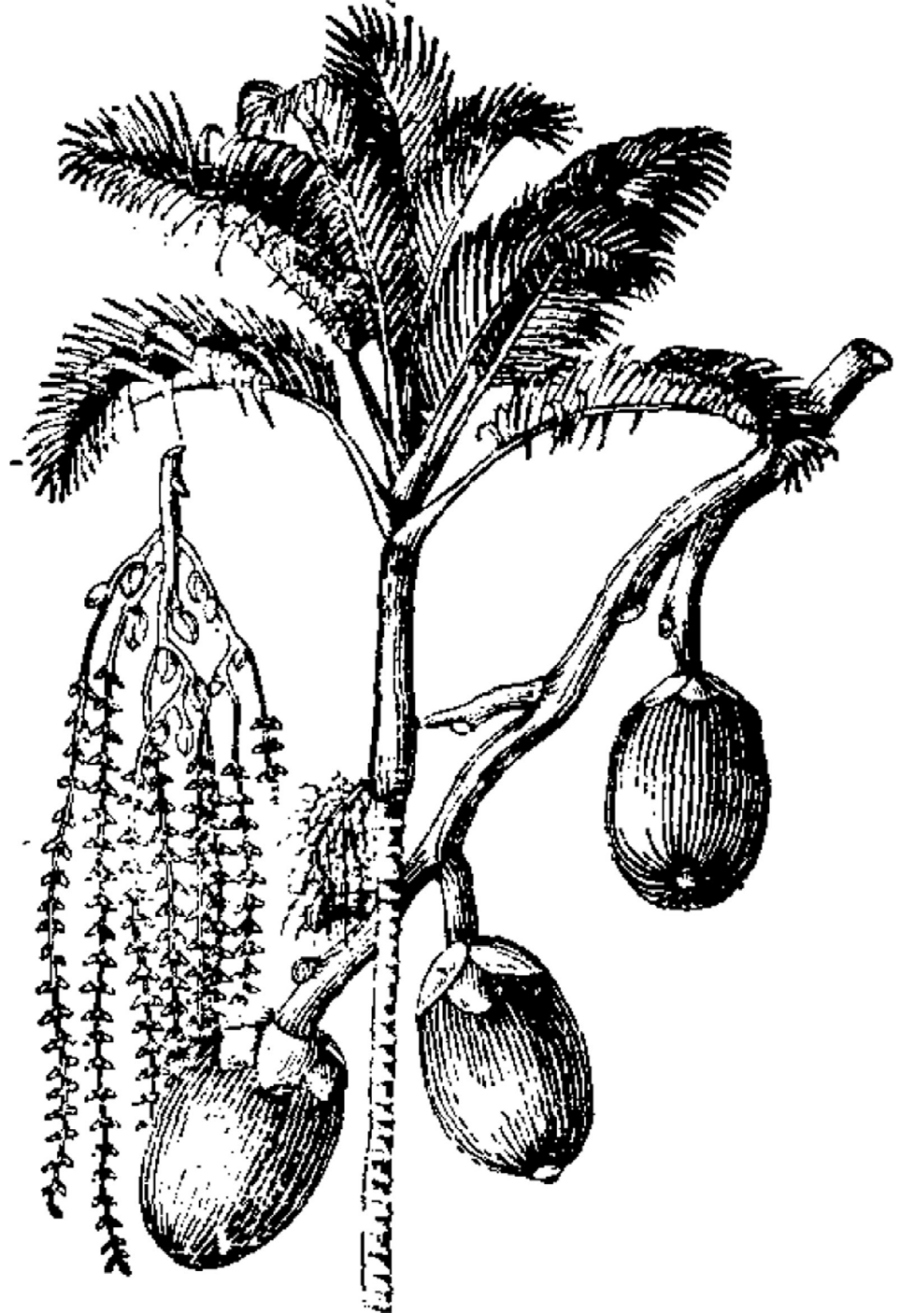
A diagrammatic illustration of betel nut. Betel nut is obtained from the betel catechu tree. Source: Iconographia cormophytorum sinicorum Tomus **Ⅴ** (1976) page: 356 (In Chinese).

Betel quid (BQ) is one of the most widely used psychoactive substances worldwide, with approximately 600 million consumers mostly located in South Asia, Southeast Asia, and the islands of the Pacific (1). Betel nut for chewing is obtained from the betel catechu tree (Figure 1). Arecaidine, arecoline, guvacine, and guvacoline are the four main alkaloids in betel nut (2). Arecoline, with parasympathomimetic properties acting on both nicotinic and muscarinic receptors, has been thought to be responsible for several effects of BQ chewing such as euphoria, palpitation, a sense of well-being, and heightened alertness (3). Arecoline induces a cardio-acceleratory response in humans and an arousal response in animals (4, 5). Moreover, arecaidine and guvacine are GABA uptake inhibitors (6). The addictive properties of BQ chewing are demonstrated by withdrawal symptoms shown by even mild habitual users (7). When BQ is chewed, a host of harmful chemicals contribute to deleterious effects (8). Mounting scientific evidence indicates a possible causal association between long-term BQ use and oral squamous cell carcinoma, complications of pregnancy, systemic diseases such as type 2 diabetes, as well as a wide range of other adverse health effects (9–11). BQ is regarded as a human carcinogen by the World Health Organization (12). It is an urgent need to understand the mechanisms of BQ dependence and develop corresponding strategies to decrease the potential dangers of BQ.

Betel quid, similar to other abused substances, are associated with a dependency syndrome including positive and negative effects and withdrawal. The positive effects include a sense of well-being, increased concentration, mild euphoria, and relaxation, postprandial satisfaction, heightened alertness, and increased capacity to work (13). Ingestion of BQ has also been associated with negative effects including habituation, addiction, and withdrawal (7). The severity of BQ withdrawal syndrome, which includes symptoms such as irritability, anxiety, mood swings, and insomnia has been compared to that of amphetamine use (14). Despite the antiquity and popularity of BQ chewing, its related and comparative effects on the neuroanatomy and function of the brain have not been investigated systematically in humans in contrast to other substances.

The emergence of sensitive high-resolution technology for neuroimaging has contributed to new insights into the neuropathology of BQ addiction. With respect to structural brain abnormalities associated with BQ chewing, Chen et al. (15) found that subjects with BQ dependence exhibited significant gray matter volume loss in the midbrain, right rostral anterior cingulate cortex (ACC), bilateral dorsolateral prefrontal cortex, and right superior temporal gyrus, as well as increased gray matter volume in the hippocampal and precuneus. In another structural study of white matter abnormalities in BQ chewers, Weng et al. (16) reported that regions including the right ACC, the midbrain, the bilateral angular gyrus, the right superior temporal gyrus, the bilateral superior occipital gyrus, the left middle occipital gyrus, the bilateral superior and inferior parietal lobule, and the bilateral postcentral and precentral gyrus were increased in BQ chewers compared to controls (16). In addition, recent functional studies of BQ chewers using functional magnetic resonance imaging (fMRI) (17) found that connectivity from the ACC to the reward network was increased and connectivity from the ACC to the default mode network (DMN) was decreased in BQ chewers compared to healthy controls (HCs). Zhu et al. (18) reported that BQ-dependent individuals had decreased functional connectivity in the anterior aspect of the DMN. Another resting-state fMRI study observed that BQ chewers exhibited a significant decrease in low-frequency fluctuation (ALFF) and regional homogeneity in the prefrontal gyrus (19).

In our prior study using independent component analysis (ICA), we found that acute effects caused by BQ may alter functional connectivity of frontal and default networks (20). ICA is a data driven, multivariate method that defines functionally connected brain regions (21, 22) by identifying spatially independent components with strong temporally coherent hemodynamic signal change over time (23, 24). ICA can be used to evaluate functional connectivity by either comparing voxel-wise spatial differences within a component or assessing temporal connectivity across pairs of spatially independent ICA components.

In this study, we used ICA to assess brain functional connectivity, focusing on naturally occurring large scale networks with fewer assumptions and less bias relative to the strategy of constraining the analysis by pre-specifying regions or seeds. We first used group ICA to identify resting state networks across all subjects including individuals with BQ dependence (BQD) and HCs, and then compared the networks between these two groups to explore global functional brain networks and identify which subsets may correspond to underlying neuropathology associated with BQ dependence. This study was conducted to elucidate the neuropathological changes that correlate with BQ dependency, which may be useful for considering treatment approaches for achieving abstinence.

## Materials and Methods

### Participants

This study was approved by the Ethics Committee of the Second Xiangya Hospital of Central South University. Written informed signed consent was provided by each participant before being included in the study. All of the participants were male. Individuals with BQD met the following inclusion criteria: (1) 18–40 years of age; (2) Han Chinese ethnicity; (3) completed nine or more years of education; (4) right-handed; (5) meeting the diagnostic criteria for BQD as persons with usage of BQ at least 1 day at a time for more than 3 years and with a score of 5 or higher on the Betel Quid Dependence Scale (BQDS). The BQDS was specifically developed by Lee et al. (25) to measure BQ dependence. The BQDS is a 16-item self-report instrument comprised of three factors: physical and psychological urgent need, increasing dose and maladaptive use. The BQDS has an optimal cut-off score of 4, optimal sensitivity of 0.926, and specificity of 0.977, and a predictive accuracy of up to 99.3%. Furthermore, the BQDS has been found to have high internal consistency (α = 0.921) and to exhibit good degrees of validity and reliability for both Chinese-speaking and English-speaking chewers (25, 26). Participants were excluded if they had: (1) a history of neurological disorder or other serious physical illness; (2) a history of any DSM 5 Axis-I mental disorders; (3) a history of substance abuse other than BQ; (4) a contraindication to MRI; or (5) a history of electroconvulsive therapy. Twenty-seven HCs were recruited from the community in the Changsha City area; all of them were persons just graduated from university who was introduced one by one to participate in our study. The HC’s inclusion and exclusion criteria were equivalent to those for patient group, with the exception of an additional requirement that controls did not meet diagnostic criteria for BQD or have a family history of psychiatric illness among their first-degree relatives. All participants did not use any psychoactive substance in the 24 h period before scanning. The Beck Depression Inventory (27) and Beck Anxiety Inventory (28) were also used to assess the emotional status of each subject before resting-state scanning.

### Images Acquisition and Preprocessing

Resting-state images were acquired on a Philips Gyroscan Achieva 3.0-T scanner in the axial direction. Gradient-echo echo planar imaging sequence was used with the following parameters as following: repetition time = 2,000 ms, echo time = 30 ms, flip angle = 90°, matrix = 64 × 64, slice thickness = 4 mm, gap = 0 mm, field of view = 24 cm × 24 cm, number of slices = 36. Earplugs were used to minimize scanner noise, and foam pads were used to minimize head motion. Participants were instructed to lie supine and remain at rest and motionless with their eyes closed. Each resting-state fMRI scan lasted 500 s, and a total of 250 image volumes were obtained. The Data Processing Assistant for Resting-State fMRI (DPARSF) (29) toolbox was used to the preprocess the fMRI imaging data through Statistical Parametric Mapping (SPM8^1^). The initial 10 images were discarded for scanner calibration and for participants to adjust to the scanner environment. The remaining 240 image volumes of the resting-state fMRI data were corrected by slice timing and realigned for head motion. Realignment allows for a maximum translation and/or rotation met the following two criteria (1): maximum displacement in the *x, y*, or *z* axis was less than 2 mm and (2) angular rotation about each axis was less than 2°. At first, 28 BQD and 30 HCs were scanned, but 4 BQD and 3 HCs were excluded due to excessive head movement artifacts (i.e., rotations larger than 2° or translations greater than 2 mm) during fMRI scanning. Spatial normalization to the standard Montreal Neurological Institute echo-planar imaging template in the SPM package, data were then spatially normalized into standard coordinates and resampled to 3 mm × 3 mm × 3 mm voxels (30). Finally, we smoothed the resampled images with a Gaussian kernel (full width at half-maximum, 8 mm).

### Independent Component Analysis

The GIFT group ICA toolbox^2^ was used to determine temporally distinct components in 51 fMRI scans of all individuals including 24 BQD and 27 HCs. The exact pipeline for ICA has been applied in our prior works (20, 31, 32) and other studies (23, 33). A modified minimum description length algorithm (34, 35) that accounts for spatial correlation (35) was used to calculate the dimension estimation to determine the number of components. The average number of independent components was 26, estimated from data across all subjects. ICASSO software based on a random initiation method was used to investigate the stability of the derived networks (36). Principal component analysis (23) was then used to reduce the dimensions of the functional data, followed by independent component estimation that produced spatial maps and time courses with the infomax algorithm (24). A method based on principal component analysis compression and projection (23, 37) was used to back-reconstruct the independent components’ spatial maps and time courses for each subject and image distribution was centered to a mode of 0 (38). The specific back-reconstruction feature of the GIFT algorithm allows analysis of all participants simultaneously as part of a large ICA group matrix (37). For each IC, the time courses of each component, therefore, represented a pattern of synchronized brain activity, whose coherency pattern across voxels was represented in the associated spatial map. To display voxels relevant to a particular IC, the intensity values in each map were converted to *z* values (38).

### Identifying Networks

**Figure 2:**
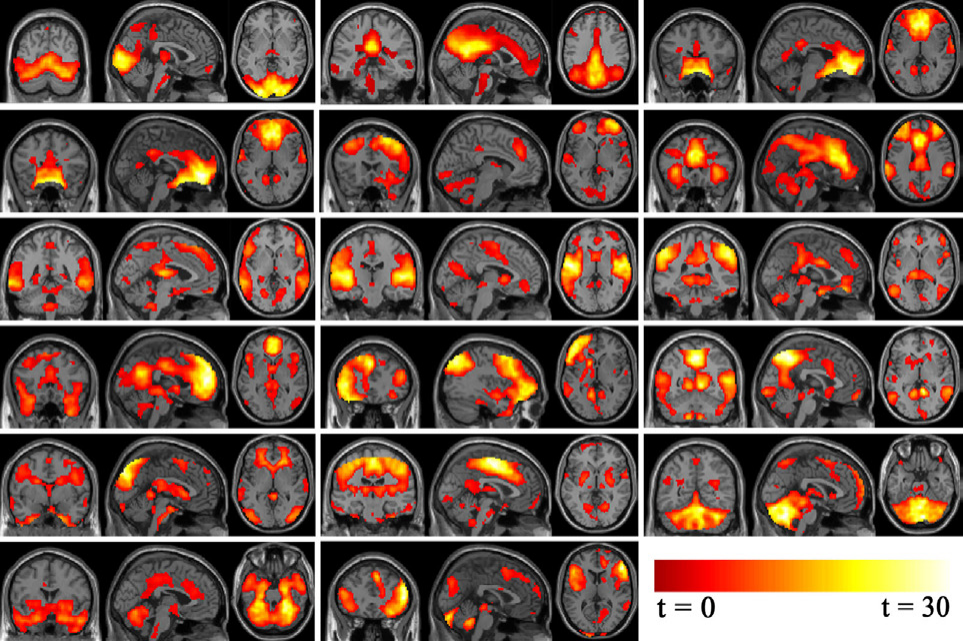
Independent component analysis non-noise resting state networks from left to right (top row: occipital/temporal, posterior default mode, Visual. Second row: orbitofrontal, right frontoparietal, anterior default mode. Third row: frontotemporal, temporal, parietal. Fourth row: medial frontal/anterior cingulated, left frontoparietal, occipital/parietal. Fifth row: occipital/temporal/cerebellum, frontotemporal/paralimbic, frontotemporal/cerebellum. Bottom row: cerebellum/midbrain, temporal/limbic.).

Standard methods of rejecting artifacts were employed to identify valid resting-state networks. Components were examined visually to eliminate obvious artifacts, then spatially correlated to *a priori* probabilistic gray matter, white matter, and cerebrospinal fluid templates (in SPM8) using multiple regressions. Components having low association (|beta| < 0.3) with gray matter template and high association (|beta| > 2) with white matter and cerebrospinal fluid template were regarded as artifacts. Statistical maps for each remaining component were created using a voxel-wise one-sample *t*-test at group level, with a threshold of *p* < 0.05 and false discovery rate (FDR) correction to further examine validity. Nine components were discarded as noise and 17 components were deemed valid networks for further analysis as illustrated in Figure 2. The probability function of each network was determined according to the methods of Laird et al. (39) and Khadka et al. (40).

### Statistical Analysis

Statistical analyses of participant demographic and clinical characteristics were carried out using the Statistical Package for the Social Sciences 19.0 (IBM SPSS Inc., USA). Comparisons of demographic variables were made using independent-sample *t*-tests for continuous variables.

The two sample *t*-test was used to explore differences in each component between BQD and HC using SPM8.^3^ Since age and education have been reported to impact brain structure and functions (41, 42), these two variables were examined as covariates in our analysis in SPM8 to rule out their effects in our functional results. Results were considered significant at *p* < 0.001 uncorrected and cluster size = 20, for statistical maps that did not survive FDR correction. The final statistical parametric map represented the functional connectivity differences in each network between BQD and HCs.

### Correlation Analysis

To investigate the association between the functional connectivity of the networks and BQDS scores, Spearman correlation was calculated. The networks showing significantly altered functional connectivity between the BQD and healthy control groups were extracted as regions of interest. Correlation analysis was implemented to the mean value of the functional connectivity in regions of interest and BQDS scores by using SPSS 19.0. In addition, we were interested in whether age and years of education were related to the altered functional connectivity, so we determined correlation coefficients between the functional connectivity in the regions of interest and years of age and education in the BQD group.

## Results

### Demographic and Clinical Characteristics

All the 51 people in the sample (24 BQD users and 27 HCs) were male. The mean age of the BQD sample was 23.54 ± 3.87 years; the mean age of the HCs was 24.52 ± 1.45. The BQD sample had completed 15.13 ± 1.73 years education and the HC individuals had completed 16.00 ± 0.00 years education. Individuals in the BQD group indicated that they had been chewing BQ with dependency syndrome for a mean duration of 7.75 ± 4.28 years (range 3.25–18 years). The average score of BQDS in the BQD group was 7.42 ± 1.86. The scores on the Beck Depression Inventory (mean score 10.58 ± 6.69, range 0–24 in the BQD group; mean score 3.89 ± 4.63, range 0–14 in the HC group) and Beck Anxiety Inventory (mean score 28.50 ± 6.20, range 21–45 in the BQD group; mean score 23.19 ± 2.66, range 21–32 in the HC group) showed that none of the participants had depression or anxiety. There was a difference in years of education between the BQD and HC groups; age and years of education were examined as covariates in our resting-state imaging analysis. The demographics of BQD and HC participants are summarized in Table 1.

**Table 1:** Demographics and clinical characteristic of participants.

	Betel quid dependent (mean ± SD)	HC (mean ± SD)	*t*	*p-*Value
Age (years)	23.54 (3.87)	24.52 (1.45)	−1.22^a^	0.23
Gender (male/female)	24/0	27/0		
Education (years)	15.13 (1.73)	16.00 (0.00)	−2.64^a^	0.01*
Betel Quid Dependence Scale	7.42 (1.86)	N/A		
Duration of BQ (years)	7.75 (4.28)	N/A		
Beck Depression Inventory	10.58 (6.69)	3.89 (4.63)	4.20^a^	0.00*
Beck Anxiety Inventory	28.50 (6.20)	23.19 (2.66)	4.06^a^	0.00*

*N/A, not applicable*.

*^a^Independent-samples t-test*.

*p < 0.05.

### Network Connectivity Differences between BQD and HC

**Figure 3:**
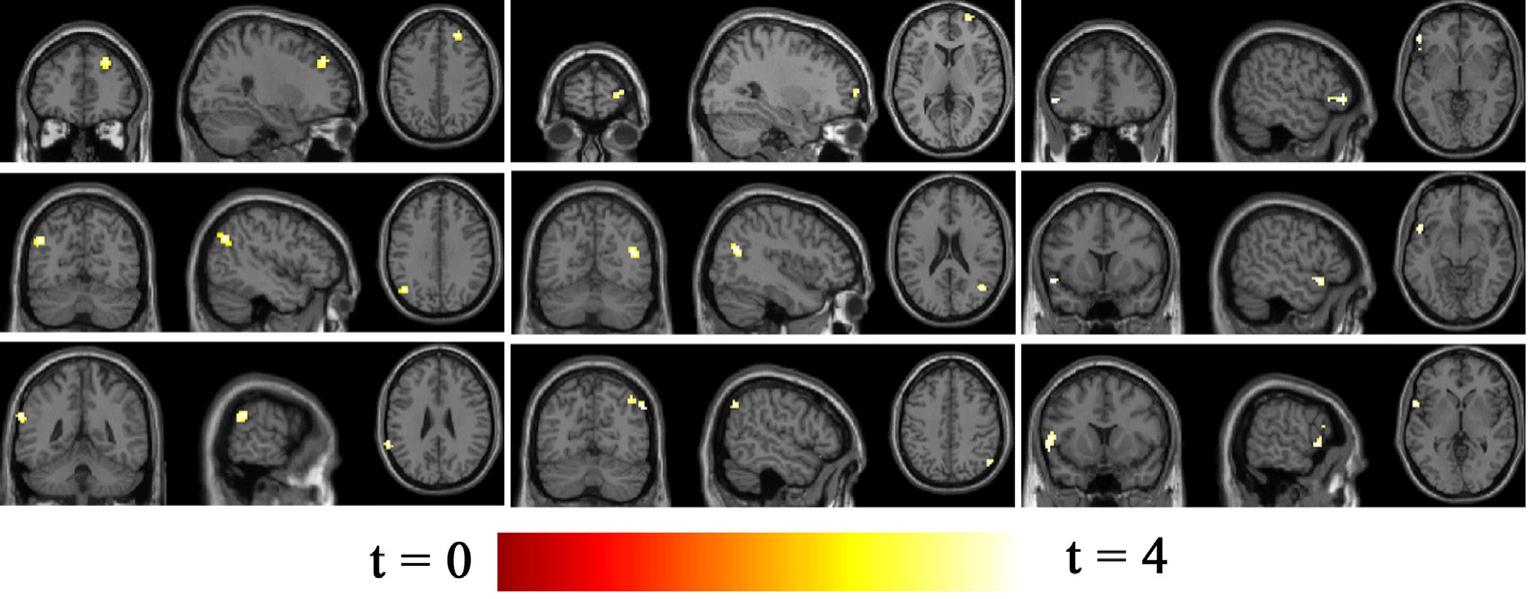
Differences in network connectivity in participants between betel quid (BQ) dependence and healthy control, determined through two sample *t*-tests (*p* < 0.001 uncorrected). Increased connectivity networks in participants with BQ dependence from left to right (top row: orbitofrontal, right frontoparietal, frontotemporal. Second row: left frontoparietal, occipital/parietal, frontotemporal/cerebellum. bottom row, first one: temporal/limbic) and decreased networks from left to right (bottom row, second and third: parietal and medial frontal/anterior cingulated). The color bars represent the range of *t* value.

Seventeen resting-state functional networks were validated (Table 2; Figure 2). Functional connectivity differences between BQD and HC groups were found in nine networks. Compared with HCs, BQD individuals exhibited increased connectivity in orbitofrontal, right frontoparietal, frontotemporal, left frontoparietal, occipital/parietal, frontotemporal/cerebellum, and temporal/limbic networks, and decreased connectivity in the parietal and medial frontal/anterior cingulate networks (two sample *t*-tests, *p* < 0.001 uncorrected, Table 2; Figure 3).

**Table 2:** Identified networks and their connectivity differences between BQ dependence and healthy control (two sample *t*-test, *p* < 0.001 uncorrected).

IC number	Network	Direction	Cluster size	Region location (AAL)	Brodman area	MNI		
						*X*	*Y*	*Z*
1	Noise	–	–	–	–	–		
2	Noise	–	–	–	–	–		–
3	Noise	–	–	–	–	–		–
4	Occipital/temporal	Negative	–	–	–	–		–
5	Posterior default mode	Negative	–	–	–	–		–
6	Visual	Negative	–	–	–	–		–
7	Orbitofrontal	BQD > HC	35	Frontal_Mid_R, Frontal_Sup_R	9	27	42	39
8	Noise	–	–	–	–	–		
9	Right frontoparietal	BQD > HC	21	Frontal_Sup_R	10	30	66	6
10	Anterior default mode	Negative	–	–	–	–		
11	Frontotemporal	BQD > HC	22	Frontal_Inf_Tri_L, Frontal_Inf_Orb_L	47, 10	−51	36	−3
12	Temporal	Negative	–	–	–	–		
13	Parietal	BQD < HC	43	Angular_R	7, 39, 40	54	−66	42
14	Medial frontal/anterior cingulate	BQD < HC	57	Frontal_Inf_Oper_L, Frontal_Inf_Tri_L, Temporal_Sup_L	44, 22	−60	12	0
15	Noise	–	–	–	–	–		
16	Noise	–	–	–	–	–		
17	Left frontoparietal	BQD > HC	40	Angular_L	39	−45	−66	33
18	Occipital/parietal	BQD > HC	34	Temporal_Mid_R, Occipital_Mid_R, Angular_R	39	42	−63	21
19	Occipital/temporal/cerebellum	Negative	–	–	–	–		
20	Frontotemporal/paralimbic	Negative	–	–	–	–		
21	Noise	–	–	–	–	–		
22	Frontotemporal/cerebellum	BQD > HC	20	Temporal_Pole_Sup_L	38	−51	12	−9
23	Noise	–	–	–	–	–		
24	Noise	–	–	–	–	–		
25	Cerebellum/midbrain	Negative	–	–	–	–		
26	Temporal/limbic	BQD > HC	26	SupraMarginal_L, Temporal_Sup_L	40	−63	−45	27

*IC, independence component; AAL, anatomical automatic labeling; MNI, Montreal Neurological Institute; BQD, betel quid dependence; HC, healthy control; Negative, no statistical significance; L/R, left/right; Sup, superior; Mid, middle; Inf, inferior; Tri, triangularis; Orb, orbital; Oper, operculum*.

### Correlation Analysis

Correlation analysis revealed that the BQDS scores were positively related to the increased functional connectivity in the orbitofrontal (*r* = 0.39 *p* = 0.03, Table 3) while negatively related to the decreased functional connectivity in medial frontal/anterior cingulate networks (*r* = −0.35 *p* = 0.02, Table 3) in the BQD individuals. No significant correlations were observed between years of age or education and the functional connectivity of the altered networks in the BQD group (*p* > 0.05).

**Table 3:** Spearman’s correlation coefficients between functional connectivity and betel quid dependence scale (BQDS) scores in betel quid dependence individuals (*n* = 24, *p < 0.05 is statistically significant).

Functional connectivity	BQDS scores	
	*r*	*p*
Orbitofrontal	0.39	0.03*
Right frontoparietal	0.15	0.48
Frontotemporal	0.22	0.31
Parietal	0.14	0.51
Medial frontal/anterior cingulate	−0.35	0.02*
Left frontoparietal	−0.31	0.14
Occipital/parietal	0.09	0.69
Frontotemporal/cerebellum	0.14	0.51
Temporal/limbic	−0.15	0.49

## Discussion

The current study used ICA to identify which brain networks were affected and how functional connectivity was changed by chronic BQ chewing. The resting-state networks identified in the present study were consistent with previous multivariate fMRI data decompositions that adopted ICA (21, 43). Nine of these networks differed between HCs and BQ chewers, with increased connectivity in orbitofrontal, frontotemporal, bilateral frontoparietal, occipital/parietal, frontotemporal/cerebellum, and temporal/limbic networks, and decreased connectivity in the parietal and medial frontal/anterior cingulate networks in BQ chewers. Interestingly, increased connectivity in the orbitofrontal, frontotemporal, and bilateral frontoparietal networks and decreased connectivity in the medial frontal/anterior cingulated network are consistent with our previous findings (20), showing similar changes in these networks associated with acute effects of BQ chewing. Furthermore, the correlation between the BQDS scores and functional connectivity in the orbitofrontal and medial frontal/anterior cingulate networks in the BQD individuals giving more solid evidence for altered functional connectivity networks in BQ chewers.

Most of the networks functional connectivity associated with BQ chronic chewing are related to frontal networks. The orbitofrontal network plays a critical role in dopaminergic reward sensitivity and decision-making (44). It receives direct and indirect (*via* the thalamus) dopaminergic projections from the nucleus accumbens, ventral tegmental area, and other limbic brain regions such as the hippocampus, amygdale, and cingulate gyrus. In turn, there are dense projections from the orbitofrontal region to the nucleus accumbens and these limbic regions. This makes the orbitofrontal cortex a highly relevant region within theoretical addiction circuitry. For instance, a recent study reports that the orbitofrontal region may underlie neural sensitivity to cannabis cues (45). Moreover, brain imaging results showing that the orbitofrontal cortex is activated by cues or acute use of drug in addicts (46) support our findings of increased orbitofrontal connectivity in BQ chewers.

Increased functional connectivity in the frontotemporal, bilateral frontoparietal, and occipital/parietal networks in BQ chewers were also found in this study. These results are similar to other recent findings (16) showing increased diffusion anisotropy in the temporal gyrus, the bilateral superior and inferior parietal lobule, and occipital gyrus. The temporal gyrus may be involved in auditory processing (47), perception of facial emotions (48), and social cognition (49). Moreover, the frontotemporal network is involved with the frontoparietal network in the development of language comprehension (50), and these cognitive abilities and behaviors are reported to be impaired in many BQ chewers (51). Increased connectivity in the occipital/parietal network, which is involved in visuospatial processing (52), may contribute to the findings that BQ chewing can immediately facilitate the dependent chewers’ visuospatial processing (53).

Decreased functional connectivity was found in the medial frontal/anterior cingulate network in BQ chewers compared to HCs. A recent BQ resting-state neuroimaging study also found decreased functional connectivity in the ventral medial prefrontal cortex (18). Neuroimaging studies have found that addicted subjects exhibited a reduction of dopamine D2 receptor in striatal (54, 55), which is associated with reduced activity of the medial frontal and ACC. Interestingly, one neurochemical study found that in the bilateral anterior cingulated cortex, *N*-acetyl-aspartate/creatine (NAA/Cr) were lower and choline (Cho)/Cr and glutamate + glutamine (Glx)/Cr were higher in BQD individuals compared to the HCs, but an increase was found for myoinositol (mI)/Cr in BQD individuals only in the left anterior cingulated cortex (56) indicating neurotransmitter or metabolic dysregulation related to BQD and providing new evidence for dysfunction in anterior cingulated cortex in BQD. Moreover, Ma et al. (57) observed that heroin users have decreased functional connectivity in the right hippocampus and the left caudate in the DMN. The DMN is more active when the brain is at baseline and reliably deactivated when individuals focus on the external environment or on performing goal-directed tasks (58). Greicius et al. (59) demonstrated that major depression is associated with increased resting-state functional connectivity in the medial frontal/anterior cingulated cortex. The medial and frontal/anterior cingulated cortices are important components of the DMN associated with resting-state brain function (60). Therefore, the effects associated with BQ chewing such as reduced mind-wandering, better sustained attention, ameliorated depression, and improved social cognition (13, 61, 62) may result from the suppressive functional connectivity of the DMN.

Decreased functional connectivity was also found in the parietal network in BQ chewers compared to HCs. Weng et al. (16) found increased diffusion anisotropy in the inferior parietal lobule in BQ chewers. However, decreased long-range functional connectivity density in the bilateral inferior parietal lobule was observed in BQ chewers relative to HCs (63). The parietal cortex is related to cognition and behaviors including verbal working memory, verbal fluency, learning and acquisition skills, and complex sequential motor behavior (64). A neuropsychological study (65) demonstrated that cocaine-dependent patients have poorer performance related to executive functions, memory functions, and verbal memory and learning than HCs. We speculate that the dysfunction of functional connectivity in the parietal network is also associated with changes in social cognition and behaviors in BQ chewers, although neuropsychological evaluations were not performed in our study.

There are some limitations in this study. First, uncorrected *p* < 0.001 levels were used in the statistical analysis, which increases the risk of type I errors; therefore, this study should be considered exploratory in nature. Second, our results could be possibly confounded by the use of other substances, especially as cigarettes, although all the recruited individuals conformed to the inclusion and exclusion criteria. Third, we did not conduct neuropsychological evaluations with our subjects to identify BQ-dependent patients, which restricted our interpretation of the findings. Finally, a relationship may exist between brain functional connectivity and craving to BQ and the time since last BQ use; however, neither of them was assessed, which would be a potentially confounding factor to this study.

In conclusion, using ICA, we found both increased connectivity (including orbitofrontal, frontotemporal, bilateral frontoparietal, occipital/parietal, frontotemporal/cerebellum, and temporal/limbic networks) and decreased connectivity (parietal and medial frontal/anterior cingulate networks) in BQ chewers compared to HCs. Additionally, the BQDS scores correlated with the functional connectivity in the orbitofrontal and medial frontal/anterior cingulate networks in the BQD individuals. Altered functional connectivity in these networks may play a key role in BQ’s psychological and physiological effects. This study gave us an opportunity to understand the neurophysiology associated with BQ dependence.

## Ethics Statement

This study was approved by the Ethics Committee of the Second Xiangya Hospital of Central South University. Written informed signed consent was provided by each participant before being included in the study.

## Author Contributions

The manuscript was authored by XH, WP, HL, XL, AG, SD, ZX, and ZL. XH wrote the first draft of the manuscript. All authors have personally reviewed the manuscript and gave final approval of the version attached.

## Conflict of Interest Statement

The authors declare that the research was conducted in the absence of any commercial or financial relationships that could be construed as a potential conflict of interest.

## Footnotes

^1^http://www.fil.ion.ucl.ac.uk/spm.

^2^http://icatb.sourceforge.net/, version 3.0a.

^3^http://www.fil.ion.ucl.ac.uk/spm.

## References

[1] Boucher BJ Mannan N. Metabolic effects of the consumption of *Areca catechu*. Addict Biol (2002) 7:103–10. 10.1080/13556210120091464 11900629

[2] Zhang W Wei J Chen W Zhang H The chemical composition and phenolic antioxidants of areca (areca catechu L) seeds. International Conference on Agricultural and Biosystems Engineering, Advances in Biomedical Engineering Hong Kong (2011). p. 16–22.

[3] Von Euler US Domeij B Nicotine-like actions of arecoline. Acta Pharmacol Toxicol (Copenh) (1945) 1:263–9. 10.1111/j.1600-0773.1945.tb02581.x 21028316

[4] Abramson LB Brown AJ Sitaram N. A cardioacceleratory response to low-dose arecoline infusion during sleep in patients with major depressive disorder: relationship to REM sleep induction. Psychiatry Res (1985) 16:189–98. 10.1016/0165-1781(85)90106-4 4089053

[5] Rinaldi F Himwich HE Alerting responses and actions of atropine and cholinergic drugs. AMA Arch Neurol Psychiatry (1955) 73:387–95. 10.1001/archneurpsyc.1955.02330100019005 14360856

[6] Lodge D Johnston GA Curtis DR Brand SJ. Effects of the *Areca* nut constituents arecaidine and guvacine on the action of GABA in the cat central nervous system. Brain Res (1977) 136:513–22. 10.1016/0006-8993(77)90075-0 922499

[7] Pickwell SM Schimelpfening S Palinkas LA. ’Betelmania’. Betel quid chewing by Cambodian women in the United States and its potential health effects. West J Med (1994) 160:326–30. 8023480 PMC1022421

[8] Dasgupta R Saha I Pal S Bhattacharyya A Sa G Nag TC Immunosuppression, hepatotoxicity and depression of antioxidant status by arecoline in albino mice. Toxicology (2006) 227:94–104. 10.1016/j.tox.2006.07.016 16945459

[9] Trivedy CR Craig G Warnakulasuriya S. The oral health consequences of chewing *Areca* nut. Addict Biol (2002) 7:115–25. 10.1080/13556210120091482 11900631

[10] Yang MS Chang FT Chen SS Lee CH Ko YC. Betel quid chewing and risk of adverse pregnancy outcomes among aborigines in southern Taiwan. Public Health (1999) 113:189–92. 10.1038/sj.ph.1900563 10483082

[11] Mannan N Boucher BJ Evans SJ. Increased waist size and weight in relation to consumption of *Areca catechu* (betel-nut); a risk factor for increased glycaemia in Asians in east London. Br J Nutr (2000) 83:267–75. 10.1017/S0007114500000349 10884715

[12] IARC Working Group on the Evaluation of Carcinogenic Risks to Humans. Betel-quid and *Areca*-nut chewing and some *Areca*-nut derived nitrosamines. IARC Monogr Eval Carcinog Risks Hum (2004) 85:1–334. 15635762 PMC4781453

[13] Chu NS. Neurological aspects of *Areca* and betel chewing. Addict Biol (2002) 7:111–4. 10.1080/13556210120091473 11900630

[14] Giri S Idle JR Chen C Zabriskie TM Krausz KW Gonzalez FJ. A metabolomic approach to the metabolism of the *Areca* nut alkaloids arecoline and arecaidine in the mouse. Chem Res Toxicol (2006) 19:818–27. 10.1021/tx0600402 16780361 PMC1482804

[15] Chen F Zhong Y Zhang Z Xu Q Liu T Pan M Gray matter abnormalities associated with betel quid dependence: a voxel-based morphometry study. Am J Transl Res (2015) 7:364–74. 25901203 PMC4399099

[16] Weng JC Kao TW Huang GJ Tyan YS Tseng HC Ho MC. Evaluation of structural connectivity changes in betel-quid chewers using generalized q-sampling MRI. Psychopharmacology (Berl) (2017) 234(13):1945–55. 10.1007/s00213-017-4602-0 28342092

[17] Liu T Li J Zhao Z Zhong Y Zhang Z Xu Q Betel quid dependence is associated with functional connectivity changes of the anterior cingulate cortex: a resting-state fMRI study. J Transl Med (2016) 14:33. 10.1186/s12967-016-0784-1 26837944 PMC4736480

[18] Zhu X Zhu Q Jiang C Shen H Wang F Liao W Disrupted resting-state default mode network in betel quid-dependent individuals. Front Psychol (2017) 8:84. 10.3389/fpsyg.2017.00084 28194128 PMC5276995

[19] Liu T Li JJ Zhao ZY Yang GS Pan MJ Li CQ Altered spontaneous brain activity in betel quid dependence: a resting-state functional magnetic resonance imaging study. Medicine (Baltimore) (2016) 95:e2638. 10.1097/MD.0000000000002638 26844480 PMC4748897

[20] Huang X Liu Z Mwansisya TE Pu W Zhou L Liu C Betel quid chewing alters functional connectivity in frontal and default networks: a resting-state fMRI study. J Magn Reson Imaging (2017) 45:157–66. 10.1002/jmri.25322 27227967

[21] Allen EA Erhardt EB Damaraju E Gruner W Segall JM Silva RF A baseline for the multivariate comparison of resting-state networks. Front Syst Neurosci (2011) 5:2. 10.3389/fnsys.2011.00002 21442040 PMC3051178

[22] Calhoun VD Adali T. Multisubject independent component analysis of fMRI: a decade of intrinsic networks, default mode, and neurodiagnostic discovery. IEEE Rev Biomed Eng (2012) 5:60–73. 10.1109/RBME.2012.2211076 23231989 PMC4433055

[23] Calhoun VD Adali T Pearlson GD Pekar JJ A method for making group inferences from functional MRI data using independent component analysis. Hum Brain Mapp (2001) 14:140–51. 10.1002/hbm.1048 11559959 PMC6871952

[24] Calhoun VD Adali T Pearlson GD Pekar JJ. Spatial and temporal independent component analysis of functional MRI data containing a pair of task-related waveforms. Hum Brain Mapp (2001) 13:43–53. 10.1002/hbm.1024 11284046 PMC6871956

[25] Lee CY Chang CS Shieh TY Chang YY. Development and validation of a self-rating scale for betel quid chewers based on a male-prisoner population in Taiwan: the Betel Quid Dependence Scale. Drug Alcohol Depend (2012) 121:18–22. 10.1016/j.drugalcdep.2011.07.027 21955360

[26] Herzog TA Murphy KL Little MA Suguitan GS Pokhrel P Kawamoto CT. The Betel Quid Dependence Scale: replication and extension in a Guamanian sample. Drug Alcohol Depend (2014) 138:154–60. 10.1016/j.drugalcdep.2014.02.022 24629627 PMC4010585

[27] Beck AT Ward CH Mendelson M Mock J Erbaugh J An inventory for measuring depression. Arch Gen Psychiatry (1961) 4:561–71. 10.1001/archpsyc.1961.01710120031004 13688369

[28] Beck AT Epstein N Brown G Steer RA An inventory for measuring clinical anxiety: psychometric properties. J Consult Clin Psychol (1988) 56:893–7. 10.1037/0022-006X.56.6.893 3204199

[29] Chao-Gan Y Yu-Feng Z DPARSF: a MATLAB toolbox for “Pipeline” Data Analysis of Resting-State fMRI. Front Syst Neurosci (2010) 4:13 10.3389/fnsys.2010.00013 20577591 PMC2889691

[30] Ashburner J Friston KJ Unified segmentation. Neuroimage (2005) 26:839–51. 10.1016/j.neuroimage.2005.02.018 15955494

[31] Zhu X Wang X Xiao J Liao J Zhong M Wang W Evidence of a dissociation pattern in resting-state default mode network connectivity in first-episode, treatment-naive major depression patients. Biol Psychiatry (2012) 71:611–7. 10.1016/j.biopsych.2011.10.035 22177602

[32] Zhou L Pu W Wang J Liu H Wu G Liu C Inefficient DMN suppression in schizophrenia patients with impaired cognitive function but not patients with preserved cognitive function. Sci Rep (2016) 6:21657. 10.1038/srep21657 26882844 PMC4756363

[33] Meda SA Gill A Stevens MC Lorenzoni RP Glahn DC Calhoun VD Differences in resting-state functional magnetic resonance imaging functional network connectivity between schizophrenia and psychotic bipolar probands and their unaffected first-degree relatives. Biol Psychiatry (2012) 71:881–9. 10.1016/j.biopsych.2012.01.025 22401986 PMC3968680

[34] Jafri MJ Pearlson GD Stevens M Calhoun VD. A method for functional network connectivity among spatially independent resting-state components in schizophrenia. Neuroimage (2008) 39:1666–81. 10.1016/j.neuroimage.2007.11.001 18082428 PMC3164840

[35] Li YO Adali T Calhoun VD. Estimating the number of independent components for functional magnetic resonance imaging data. Hum Brain Mapp (2007) 28:1251–66. 10.1002/hbm.20359 17274023 PMC6871474

[36] Himberg J Hyvarinen A Esposito F. Validating the independent components of neuroimaging time series via clustering and visualization. Neuroimage (2004) 22:1214–22. 10.1016/j.neuroimage.2004.03.027 15219593

[37] Erhardt EB Rachakonda S Bedrick EJ Allen EA Adali T Calhoun VD. Comparison of multi-subject ICA methods for analysis of fMRI data. Hum Brain Mapp (2011) 32:2075–95. 10.1002/hbm.21170 21162045 PMC3117074

[38] Stevens MC Kiehl KA Pearlson GD Calhoun VD. Brain network dynamics during error commission. Hum Brain Mapp (2009) 30:24–37. 10.1002/hbm.20478 17979124 PMC2669663

[39] Laird AR Fox PM Eickhoff SB Turner JA Ray KL McKay DR Behavioral interpretations of intrinsic connectivity networks. J Cogn Neurosci (2011) 23:4022–37. 10.1162/jocn_a_00077 21671731 PMC3690655

[40] Khadka S Meda SA Stevens MC Glahn DC Calhoun VD Sweeney JA Is aberrant functional connectivity a psychosis endophenotype? A resting state functional magnetic resonance imaging study. Biol Psychiatry (2013) 74:458–66. 10.1016/j.biopsych.2013.04.024 23746539 PMC3752322

[41] Carne RP Vogrin S Litewka L Cook MJ. Cerebral cortex: an MRI-based study of volume and variance with age and sex. J Clin Neurosci (2006) 13:60–72. 10.1016/j.jocn.2005.02.013 16410199

[42] Farthing JP Krentz JR Magnus CR Barss TS Lanovaz JL Cummine J Changes in functional magnetic resonance imaging cortical activation with cross education to an immobilized limb. Med Sci Sports Exerc (2011) 43:1394–405. 10.1249/MSS.0b013e318210783c 21266927

[43] Damoiseaux JS Rombouts SA Barkhof F Scheltens P Stam CJ Smith SM Consistent resting-state networks across healthy subjects. Proc Natl Acad Sci U S A (2006) 103:13848–53. 10.1073/pnas.0601417103 16945915 PMC1564249

[44] Volkow ND Fowler JS. Addiction, a disease of compulsion and drive: involvement of the orbitofrontal cortex. Cereb Cortex (2000) 10:318–25. 10.1093/cercor/10.3.318 10731226

[45] Filbey FM Dunlop J Ketcherside A Baine J Rhinehardt T Kuhn B fMRI study of neural sensitization to hedonic stimuli in long-term, daily cannabis users. Hum Brain Mapp (2016) 37:3431–43. 10.1002/hbm.23250 27168331 PMC5012952

[46] Schoenbaum G Shaham Y The role of orbitofrontal cortex in drug addiction: a review of preclinical studies. Biol Psychiatry (2008) 63:256–62. 10.1016/j.biopsych.2007.06.003 17719014 PMC2246020

[47] Moerel M De Martino F Formisano E. An anatomical and functional topography of human auditory cortical areas. Front Neurosci (2014) 8:225. 10.3389/fnins.2014.00225 25120426 PMC4114190

[48] Bigler ED Mortensen S Neeley ES Ozonoff S Krasny L Johnson M Superior temporal gyrus, language function, and autism. Dev Neuropsychol (2007) 31:217–38. 10.1080/87565640701190841 17488217

[49] Jou RJ Minshew NJ Keshavan MS Vitale MP Hardan AY Enlarged right superior temporal gyrus in children and adolescents with autism. Brain Res (2010) 1360:205–12. 10.1016/j.brainres.2010.09.005 20833154 PMC2990401

[50] Rodd JM Longe OA Randall B Tyler LK. The functional organisation of the fronto-temporal language system: evidence from syntactic and semantic ambiguity. Neuropsychologia (2010) 48:1324–35. 10.1016/j.neuropsychologia.2009.12.035 20038434

[51] Chu NS. Effects of betel chewing on electroencephalographic activity: spectral analysis and topographic mapping. J Formos Med Assoc (1994) 93:167–9. 7912589

[52] de Graaf TA Roebroeck A Goebel R Sack AT. Brain network dynamics underlying visuospatial judgment: an FMRI connectivity study. J Cogn Neurosci (2010) 22:2012–26. 10.1162/jocn.2009.21345 19803683

[53] Ho MC Wang CK. The effect of betel nut chewing on contour and object masking. Atten Percept Psychophys (2011) 73:2583–93. 10.3758/s13414-011-0214-7 21976155

[54] Koob GF. Neural mechanisms of drug reinforcement. Ann N Y Acad Sci (1992) 654:171–91. 10.1111/j.1749-6632.1992.tb25966.x 1632582

[55] Heinz A Siessmeier T Wrase J Hermann D Klein S Grusser SM Correlation between dopamine D(2) receptors in the ventral striatum and central processing of alcohol cues and craving. Am J Psychiatry (2004) 161:1783–9. 10.1176/appi.ajp.161.10.1783 15465974

[56] Liu T Li J Huang S Zhao Z Yang G Pan M Neurochemical abnormalities in anterior cingulate cortex on betel quid dependence: a 2D (1)H MRS investigation. Am J Transl Res (2015) 7:2795–804. 26885276 PMC4731676

[57] Ma N Liu Y Fu XM Li N Wang CX Zhang H Abnormal brain default-mode network functional connectivity in drug addicts. PLoS One (2011) 6:e16560. 10.1371/journal.pone.0016560 21298074 PMC3027699

[58] Fox MD Snyder AZ Vincent JL Corbetta M Van Essen DC Raichle ME. The human brain is intrinsically organized into dynamic, anticorrelated functional networks. Proc Natl Acad Sci U S A (2005) 102:9673–8. 10.1073/pnas.0504136102 15976020 PMC1157105

[59] Greicius MD Flores BH Menon V Glover GH Solvason HB Kenna H Resting-state functional connectivity in major depression: abnormally increased contributions from subgenual cingulate cortex and thalamus. Biol Psychiatry (2007) 62:429–37. 10.1016/j.biopsych.2006.09.020 17210143 PMC2001244

[60] Greicius MD Krasnow B Reiss AL Menon V Functional connectivity in the resting brain: a network analysis of the default mode hypothesis. Proc Natl Acad Sci U S A (2003) 100:253–8. 10.1073/pnas.0135058100 12506194 PMC140943

[61] Ho M Li R Tang T Betel nut chewing effects on sustained attention and inhibitory control after sleep deprivation. Aust J Psychol (2015) 67:222–30. 10.1111/ajpy.12081

[62] Adilijiang A Guan T Xu ZZ Hartle K Zhang YB Wang WQ The aqueous fraction of *Areca catechu* nut ameliorates demyelination in prefrontal cortex-induced depressive symptoms and cognitive decline through brain-derived neurotrophic factor-cyclic adenosine monophosphate response element-binding activation. Chin J Integr Med (2016). 10.1007/s11655-016-2455-8 27081000

[63] Liu T Li J Zhang Z Xu Q Lu G Huang S Altered long- and short-range functional connectivity in patients with betel quid dependence: a resting-state functional mri study. Cell Physiol Biochem (2016) 40:1626–36. 10.1159/000453212 28006783

[64] Witt ST Laird AR Meyerand ME. Functional neuroimaging correlates of finger-tapping task variations: an ALE meta-analysis. Neuroimage (2008) 42:343–56. 10.1016/j.neuroimage.2008.04.025 18511305 PMC2592684

[65] Cunha PJ Nicastri S Gomes LP Moino RM Peluso MA. [Neuropsychological impairments in crack cocaine-dependent inpatients: preliminary findings]. Rev Bras Psiquiatr (2004) 26:103–6. 10.1590/S1516-44462004000200007 15517061

